# Low-dose letrozole-HMG regimen reverses letrozole-induced endometrial impairment and improves frozen embryo transfer outcomes

**DOI:** 10.3389/fcell.2025.1725350

**Published:** 2025-12-16

**Authors:** Zhonglin Xiao, Feng Wang, Yali Yang, Tianxia Xiao, Jie Chen, Mengxia Li, Haizhen Liao, Chiawei Chu, Xiujun Fan, Jian V. Zhang

**Affiliations:** 1 Faculty of Data Science, City University of Macau, Macau, China; 2 Shenzhen Key Laboratory of Metabolic Health, Center for Energy Metabolism and Reproduction, Shenzhen Institutes of Advanced Technology, Chinese Academy of Sciences, Shenzhen, China; 3 Reproductive Medicine Centre, Shenzhen Hengsheng Hospital, Shenzhen, China; 4 Reproductive Center of Shenzhen Armed Police Hospital, Shenzhen, China; 5 Reproductive Medicine Department, Shenzhen Luohu People’s Hospital, Shenzhen, China; 6 Faculty of Pharmaceutical Sciences, Shenzhen University of Advanced Technology, Shenzhen, China; 7 Sino-European Center of Biomedicine and Health, Shenzhen, China

**Keywords:** infertility, endometrial receptivity, implantation, pinopode, frozen embryo transfer, letrozole, human menopausalgonadotropin, proteomics

## Abstract

**Introduction:**

Letrozole monotherapy, while effective for ovulation induction, may compromise endometrial receptivity in frozen embryo transfer (FET) cycles due to estrogen suppression. This study aimed to compare the FET outcomes and analyze the molecular mechanisms of endometrial receptivity among low-dose letrozole plus HMG (LeH) with letrozole monotherapy (Le) and natural cycles (NC).

**Methods:**

This retrospective cohort study included 5,673 infertile patients undergoing FET with one of the following protocols: LeH (n = 2,997), Le (n = 1,762), or NC (n = 914). Endometrial receptivity was assessed via serum hormone assays, scanning electron microscopy (SEM) of pinopodes on post-ovulation days 3 (D3; pre-FET) and 5 (D5; estimated implantation window), and proteomic analyses of endometrial tissue, uterine fluid, and serum on D3.

**Results:**

Clinical outcomes revealed that the LeH group had significantly higher implantation, clinical pregnancy and live birth rates compared to the Le and NC groups, especially among older women. Notably, the Le group was associated with thinner endometrium, lower estradiol levels, reduced vascularization flow index (VFI), and a lower proportion of receptive-phase endometria (28% vs. 60% in NC). In contrast, the LeH group maintained normal endometrial parameters, and resulted in a high proportion of fully developed pinopodes (84%). Proteomic profiling revealed that the Le group adversely affected processes related to cell adhesion and inflammatory regulation, while the LeH group reversed these alterations. It activated pathways important for embryo implantation and promoted an anti-inflammatory environment.

**Discussion:**

These results suggest that the LeH regimen may mitigate letrozole-induced endometrial impairment and enhances FET outcomes through structural, molecular, and immunological mechanisms, offering a promising approach for optimized endometrial preparation.

## 1 Introduction

In recent years, the popularity of frozen embryo transfer (FET) has increased substantially (De Geyter et al., 2020; Sunderam et al., 2020) owing to its lower cancellation rates and greater scheduling flexibility (Zhang et al., 2019). The neutral effect of FET on reproductive outcomes has further led to the widespread adoption of a “freeze-all” strategy in many assisted reproductive technology (ART) centers (Cobo et al., 2024; Melo et al., 2022). Within FET cycles, the adequacy of endometrial preparation is a critical determinant of success (Vinsonneau et al., 2022; Groenewoud et al., 2013), and optimizing ovulation induction protocols to enhance endometrial receptivity remains a central challenge (Xia et al., 2023).

Letrozole (Le), a third-generation aromatase inhibitor, is widely used for ovulation induction, particularly in patients with polycystic ovary syndrome (PCOS) (Bhatnagar, 2007; Castillo and Kol, 2024). Its ability to reduce estrogen levels and promote monofollicular development (Li et al., 2022) minimizes negative impacts on cervical mucus and the endometrium (Chen et al., 2023), with meta-analyses confirming significant improvements in live birth and pregnancy rates (Franik et al., 2018). However, the very mechanism that makes letrozole effective—systemic estrogen suppression—may also impair endometrial proliferation and receptivity, potentially compromising its clinical benefits in FET cycles.

Human menopausal gonadotropin (HMG), which contains both FSH and LH, is another common agent used for ovulation induction (Quaas and Legro, 2019). Although it poses risks such as ovarian hyperstimulation syndrome (OHSS) and multiple pregnancies in PCOS patients (Tummon et al., 2005; Diamond et al., 2015; Colucci et al., 2000), studies have found no significant differences in neonatal outcomes between HMG and hormone replacement treatment (HRT) cycles (Zhang Y. et al., 2023). Notably, heterogeneity in clinical pregnancy rates have been observed across different therapeutic combinations of letrozole and HMG, potentially due to variations in the dosage of letrozole and/or HMG (Xi et al., 2015; Lin et al., 2019; Zhang et al., 2021). Irrespective of these differences, these combination strategies consistently mitigate the risks of OHSS and excessive multifollicular development (Zhang et al., 2021), thereby furnishing a promising therapeutic avenue for optimizing the trade-off between efficacy and safety (Xi et al., 2015).

In our ART center, we have observed that letrozole-based cycles often present an inverse relationship between endometrial thickness and estradiol levels: adequate endometrial thickness ( ≥ 8 mm) frequently coincides with low estradiol levels ( < 200 pg/mL), and *vice versa*. This clinical pattern suggests that letrozole may indeed adversely affect endometrial preparedness, likely due to insufficient estrogenic stimulation (Bülow and Macklon, 2025).

Based on these observations, we hypothesized that supplementing letrozole with low-dose HMG could mitigate the endometrial thinning associated with letrozole monotherapy, thereby improving endometrial receptivity and overall FET outcomes. This study aims to investigate whether the LeH regimen can refine endometrial preparation and enhance the efficiency of FET cycles by counteracting the potential negative effects of letrozole on the endometrium. Through a combination of clinical, ultrastructural, and molecular analyses, we seek to provide comprehensive evidence supporting the use of LeH as a superior regimen for ovulation induction in FET.

## 2 Materials and methods

### 2.1 Study design and participants

**FIGURE 1:**
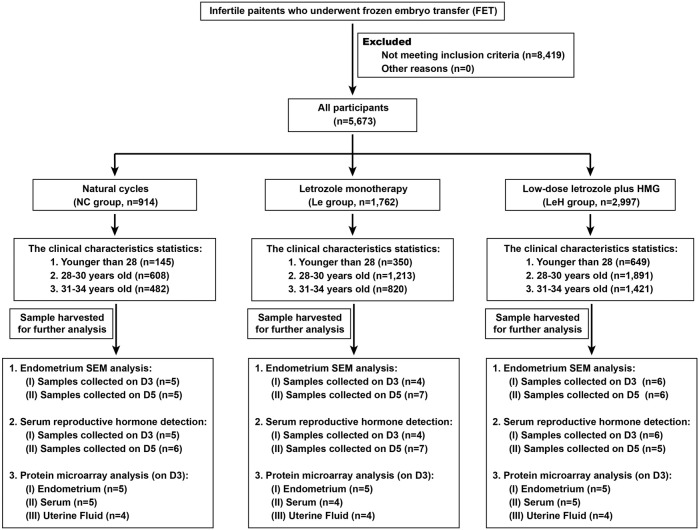
The flowchart of participants. HMG, Human Menopausal Gonadotropin; SEM, Scanning Electron Microscopy.

This retrospective cohort study analyzed data from 5,673 infertile patients undergoing FET between 2015 and 2022 at the Reproductive Center of Shenzhen Armed Police Hospital. Participants were divided into three groups based on the endometrial preparation protocol: the LeH regimen (n=2,997) , Le regimen (n=1,762) , and natural cycle (NC) group (n=914) . To ensure robust comparisons in light of the uneven sample sizes across treatment groups, all primary analyses were stratified *a priori* by female age ( < 28, 28–30, and 31–34 years). Groups were matched for age, body mass index (BMI), number of high-quality embryos transferred, endometrial thickness, estradiol levels, embryo implantation rate, clinical pregnancy rate, and live birth rate. A detailed participant selection flowchart is provided in Figure 1.

### 2.2 Endometrial preparation protocols

LeH Group: Participants received ovarian ultrasonography on the third menstrual cycle day. If no follicles ≥ 10 mm were present, low-dose letrozole (2.5 mg/day, Hengrui, China) was administered for 3–5 days, followed by HMG (75.0 U/day, Livzon, China) once follicles reached 13 mm, until follicles were ≥ 18 mm, estradiol levels exceeded 199 pg/mL, and endometrial thickness was ≥ 8 mm.

Le Group: Letrozole (2.5–5 mg/day) was given for 3–5 days if no follicle ≥ 10 mm was detected, followed by ultrasound monitoring until follicles reached ≥ 18 mm, estradiol levels were above 0.15 pg/mL, and endometrial thickness was ≥ 8 mm.

NC Group: Ovarian ultrasonography began on the 10th menstrual cycle day, continuing until follicles reached ≥ 18 mm, estradiol levels exceeded 0.15 pg/mL, and endometrial thickness was ≥ 8 mm.

For all groups, HCG (10,000 U, Livzon, China) was administered if LH levels were < 20 U/L, with embryo transfer on the fifth day post-ovulation (D5). If LH levels were ≥ 20 U/L, HCG was injected immediately, with transfer on D4. For all patients, luteal support began 2–3 days before transfer and consisted of oral dydrogesterone (20 mg, twice daily, Suwey, Belgium) combined with a vaginal progesterone soft capsule (0.2 g daily at bedtime, Cyndea, France). The medication was continued for 14 days after transfer and, upon confirmation of clinical pregnancy, maintained until the $10^{th}$ week of gestation.

### 2.3 Ethical approval

This study received approval from the Ethics Committee of the Shenzhen Institutes of Advanced Technology, Chinese Academy of Sciences (approval number: SIAT-IRB-180215-H0201) and was executed in accordance with their directives. All participants provided written informed consent (2016-1.0 version) and were enrolled in alignment with the Declaration of Helsinki.

### 2.4 Inclusion and exclusion criteria

Inclusion criteria comprised women under 35 years of age with a regular menstrual cycle (26–37 days) and at least one high-quality embryo available for transfer. Those in a fresh cycle were required to have an endometrial thickness >8 mm following ovarian stimulation. Exclusion criteria included a history of uterine surgery (e.g., curettage or hysteroscopic surgery), diagnosed gynecological conditions (e.g., ovulation disorders, adenomyosis, ovarian endometriomas, uterine fibroids, uterine abnormalities, or endometrial lesions) within 3 months prior to treatment, or cycles complicated by ovarian hyperstimulation syndrome (OHSS) or cancellation prior to embryo transfer.

### 2.5 Sample collection

Blood, uterine fluid, and endometrial tissue samples were collected from 33 participants on D3 and D5. Serum was isolated and stored at −80° C, while uterine fluid and endometrial tissues were also preserved at −80° C. A section of the endometrial tissue was set aside for H&E staining and SEM.

### 2.6 Serum hormone detection by RIA

Serum hormone levels were measured using a commercial iodine-[125I] RIA kit (Beijing North Biotechnology Institute, China) for estradiol, testosterone, progesterone, LH, FSH, and prolactin. All samples were assayed at least in triplicate. The intra-assay and inter-assay coefficients of variation were < 10% and < 15%, respectively Yang et al. (2016).

### 2.7 Histological and ultrastructural examination

Endometrial tissues were sectioned and stained with H&E for histological evaluation Xiao et al. (2025). Scanning electron microscopy (SEM) was performed to assess the ultrastructural morphology of the endometrium, with particular attention to pinopode development Zhao et al. (2021). For each sample, multiple representative fields were examined to ensure a consistent assessment.

### 2.8 Protein array analysis

Relative expression levels of 440 human cytokines in serum, uterine fluid, and endometrial tissue extracts collected on D3 were quantified using the G-Series Human Cytokine Antibody Array 440 (RayBiotech, China) Wang Q. et al. (2024).

### 2.9 Bioinformatics analysis

Fold-change (FC) and adjusted p -values for protein microarray data were calculated, using selection criteria of |log(FC)|≥1.5 and adjusted p<0.05 for bioinformatics analyses by RayBiotech, including PCA, ScatterPlot, VolcanoPlot and Heatmap Wang Q. et al. (2024). Gene Ontology (GO) and Kyoto Encyclopedia of Genes and Genomes (KEGG) pathway enrichment analyses were performed via Metascape Zhou et al. (2019). Potential protein-protein interactions (PPI) were analyzed using the STRING database and visualized with Cytoscape software Wang H. et al. (2024). Overlapping differentially expressed proteins (DEPs) were identified with the Venn tool Wang et al. (2021). Sankey diagrams and streamlined bar charts for GO and KEGG terms were generated in R Zhang et al. (2024).

### 2.10 Statistical analysis

Data were analyzed using GraphPad Prism 9.0 and SPSS 19.0, represented as mean ± SEM or mean ± standard deviation, with at least three repetitions. Unpaired Student’s t -tests were used for pairwise comparisons, with p -values < 0.05 considered significant.

## 3 Results

### 3.1 The LeH regimen improves clinical pregnancy outcomes in FET cycles

Based on reproductive physiology, patients were divided into three age groups for analysis. The clinical data revealed that the LeH group had significantly higher rates of embryo implantation, clinical pregnancy and live birth rate than the other groups, with the Le group outperforming the NC group. Notably, as age increased, these rates declined in all groups, but the LeH group’s effectiveness became more pronounced (Table 1).

**TABLE 1:** Clinical characteristics and outcomes of patients.

Parameter	Less 28 years old				28–30 years old				31–34 years old			
	NC	Le	LeH	*p-value*	NC	Le	LeH	*p-value*	NC	Le	LeH	*p-value*
	(n = 145)	(n = 350)	(n = 649)		(n = 608)	(n = 1213)	(n = 1891)		(n = 482)	(n = 820)	(n = 1421)	
Age (years)	25.45 ± 1.624	25.75 ± 1.359	25.64 ± 1.424	0.091	29.10 ± 0.825	29.04 ± 0.851	29.03 ± 0.822	0.432	32.54 ± 1.131	32.46 ± 1.116	32.44 ± 1.093	0.193
BMI $\left(kg/m^{2}\right)$	20.66 ± 2.693	20.24 ± 2.538	20.29 ± 2.392	0.75	20.29 ± 2.133	20.69 ± 2.875	20.72 ± 2.625	0.558	20.79 ± 2.367	21.06 ± 2.486	21.34 ± 2.628	0.091
	91.03%	83.71%	79.51%		88.15%	85.98%	79.61%		89.00%	85.37%	80.30%	
Embryo stage (cleavage rate)	(132/145)	(293/350)^#^	(516/649)^#^	0.003	(253/287)	(509/592)	(738/927)^#,*^	0.001	(429/482)	(700/820)	(1141/1421)^#,*^	0.001
	94.77%	95.69%	94.59%		91.78%	94.97%	93.71%		89.26%	92.82%	91.38%	
High-quality embryo rate	(290/306)	(689/720)	(1225/1295)	0.550	(558/608)	(1152/1213)^#^	(1772/1891)	0.028	(931/1043)	(1565/1686)^#^	(2734/2992)^#^	0.001
Number of high-quality embryos transferred	2.11 ± 0.393	2.06 ± 0.487	2.00 ± 0.505^#^	0.016	2.12 ± 0.502	2.05 ± 0.454^#^	2.04 ± 0.517^#^	0.059	2.16 ± 0.566	2.06 ± 0.496^#^	2.06 ± 0.525^#^	0.001
	22.76%	28.29%	46.22%		25.09%	28.89%	44.44%		30.08%	35.49%	47.01%	
Transplantation day (D4/D5)	(33/145)	(99/350)	(300/649)^#,*^	0.001	(72/287)	(171/592)	(412/927)^#,*^	0.001	(145/482)	(291/820)^#^	(668/1421)^#,*^	0.001
	46.73%	49.31%	50.50%		40.13%	46.41%	49.50%		38.26%	41.34%	44.97%	
Embryo implantation rate	(143/306)	(355/720)	(654/1295)	0.298	(244/608)	(563/1213)^#^	(936/1891)^#,*^	0.001	(399/1043)	(697/1686)	(1314/2922)^#,*^	0.027
	66.90%	72.86%	73.19%		61.67%	64.02%	71.52%		59.54%	63.54%	66.22%	
Clinical pregnancy rate	(97/145)	(255/350)	(475/649)	0.484	(177/287)	(379/592)	(663/927)^#,*^	0.001	(287/482)	(521/820)	(941/1421)^#^	0.001
	4.12%	8.24%	7.16%		8.47%	5.80%	7.24%		10.80%	9.21%	11.05%	
Abortion rate	(4/97)	(21/255)	(34/475)	0.761	(15/177)	(22/379)	(48/663)	0.025	(31/287)	(48/521)	(104/941)	0.197
	4.12%	1.96%	2.74		5.08%	2.11%	2.11%		4.53%	3.07%	2.02%	
Ectopic pregnancy rate	(4/97)	(5/255)	(13/475)	0.408	(9/177)	(8/379)	(14/663)^#^	0.476	(13/287)	(16/521)	(19/941)^#^	0.535
	56.55%	61.43%	62.10%		50.17%	53.89%	62.24%		47.93%	53.41%	54.89%	
Live birth rate	(82/145)	(215/350)	(403/649)	0.462	(144/287)	(319/592)	(577/927)^#,*^	0.001	(231/482)	(438/820))^#^	(780/1421)^#^	0.001
	42.27%	38.04%	38.74%		36.72%	47.49%	40.42%		37.63%	33.59%	38.26%	
Multiple birth rate	(41/97)	(97/255)	(184/475)	0.523	(65/177)	(180/379)^#^	(268/663))^*^	0.067	(108/287)	(175/521)	(360/941)^*^	0.064

NC, natural cycle; Le, Letrozole monotherapy; LeH, low-dose letrozole plus HMG; BMI, body mass index. Values are expressed as means ± SEM. denominators for abortion rate, ectopic pregnancy rate, and multiple pregnancy rate are based on the number of clinical pregnancies. ^#^ p<0.05 compared to the NC group; ^*^ p<0.05 compared to the Le group.

### 3.2 The LeH regimen mitigates letrozole-induced endometrial inadequacy

Our initial clinical observation indicated that letrozole monotherapy might compromise endometrial preparedness. To test this, we first compared endocrine and ultrasonographic parameters on the day of HCG administration. Consistent with letrozole’s mechanism of action, the Le group exhibited significantly reduced endometrial thickness, estradiol levels, and vascularization flow index (VFI) compared to the NC group (Table 2). Importantly, the LeH regimen prevented this decline, showing comparable endometrial thickness and VFI to the NC group, and a trend toward higher estradiol levels (Table 2), suggesting that HMG co-administration counteracts letrozole’s anti-estrogenic effects on the endometrium.

**TABLE 2:** IVF cycle clinical parameters and reproductive endocrinology metrics.

Parameter	Less 28 years old				28–30 years old				31–34 years old			
	NC	Le	LeH	*p-value*	NC	Le	LeH	*p-value*	NC	Le	LeH	*p-value*
	(n = 145)	(n = 350)	(n = 649)		(n = 608)	(n = 1213)	(n = 1891)		(n = 482)	(n = 820)	(n = 1421)	
No. of mature follicles	1.09 ± 0.281	1.25 ± 0.513	1.61 ± 1.081^#,*^	0.001	1.05 ± 0.223	1.21 ± 0.570^#^	1.53 ± 0.905^#,*^	0.001	1.09 ± 0.329	1.23 ± 0.504^#^	1.55 ± 0.965^#,*^	0.001
Endometrial thickness on the day of HCG administration	9.95 ± 1.460	9.82 ± 1.685	10.08 ± 1.820	0.173	10.20 ± 1.848	9.69 ± 1.828#	9.98 ± 1.848^*^	0.004	10.24 ± 1.836	9.57 ± 1.785^#^	9.77 ± 1.867^*^	0.001
LH level on the day of HCG administration	34.09 ± 29.717	24.63 ± 21.368^#^	17.23 ± 16.778^#,*^	0.001	31.28 ± 23.063	27.65 ± 23.991	17.00 ± 17.202^#,*^	0.001	34.67 ± 25.586	25.30 ± 21.795^#^	17.36 ± 16.195^#,*^	0.001
Estradiol level on the day of HCG administration	322.24 ± 130.722	296.84 ± 397.356	493.75 ± 434.785^#,*^	0.001	323.37 ± 203.181	307.70 ± 319.829	494.83 ± 396.801^#,*^	0.001	302.67 ± 142.928	303.03 ± 172.051^#^	502.98 ± 403.301^#,*^	0.001
Progesterone level on the day of HCG administration	1.05 ± 0.908	0.91 ± 0.737	0.84 ± 0.520^#^	0.014	1.23 ± 1.259	0.93 ± 0.658^#^	0.84 ± 0.650^#^	0.001	1.05 ± 0.771	0.88 ± 0.649^#^	0.78 ± 0.515^#,*^	0.001
Endometrial VFI	11.00 ± 0.863	10.33 ± 1.483	10.88 ± 1.822	0.555	9.82 ± 2.876	9.91 ± 1.590	10.80 ± 1.668^*^	0.029	11.01 ± 1.588	10.35 ± 1.786	10.51 ± 1.804	0.339
Uterine artery S/D	6.15 ± 0.908	5.07 ± 0.683^#^	5.71 ± 0.799^*^	0.014	5.74 ± 1.004	5.55 ± 1.115	5.59 ± 0.932	0.89	5.74 ± 0.631	5.69 ± 0.747	5.65 ± 0.892	0.854
Uterine artery PI	2.42 ± 0.319	2.20 ± 0.322	2.35 ± 0.296	0.21	2.34 ± 0.427	2.34 ± 0.421	2.27 ± 0.346	0.668	2.37 ± 0.295	2.24 ± 0.228	2.31 ± 0.313	0.276
Uterine artery RI	0.83 ± 0.026	0.80 ± 0.026^#^	0.82 ± 0.028^*^	0.033	0.82 ± 0.034	0.80 ± 0.876	0.81 ± 0.038	0.203	0.82 ± 0.020	0.82 ± 0.022	0.82 ± 0.033	0.841
Uterine artery S/D	5.96 ± 1.178	5.63 ± 0.935	5.71 ± 0.902	0.85	5.50 ± 0.743	5.80 ± 0.988	5.68 ± 0.920	0.749	5.66 ± 0.644	5.75 ± 0.889	5.60 ± 1.025	0.695
Uterine artery PI	2.46 ± 0.424	2.33 ± 0.387	2.34 ± 0.301	0.808	2.24 ± 0.222	2.38 ± 0.384	2.33 ± 0.342	0.622	2.29 ± 0.339	2.29 ± 0.337	2.30 ± 0.375	0.968
Uterine artery RI	0.83 ± 0.040	0.82 ± 0.031	0.82 ± 0.028	0.885	0.82 ± 0.024	0.82 ± 0.041	0.82 ± 0.341	0.954	0.82 ± 0.021	0.82 ± 0.032	0.81 ± 0.036	0.43

NC, natural cycle; Le, Letrozole monotherapy; LeH, low-dose letrozole plus HMG; LH, luteinizing hormone; VFI, vascularization flow index; S/D, systolic/diastolic ratio; PI, pulsatility index; RI, resistance index. Values are expressed as means ± SEM. ^#^ p<0.05 compared to the NC group; ^*^ p<0.05 compared to the Le group.

**FIGURE 2:**
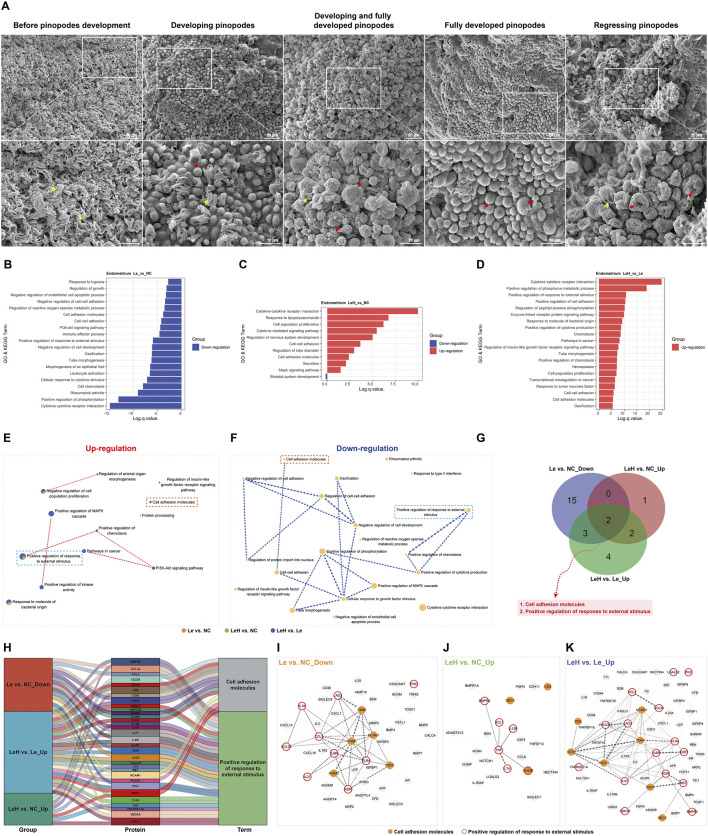
Integrated assessment of endometrial receptivity in Le/LeH/NC groups. Ultrastructural dynamics of pinopodes analyzed by SEM on D3 (pre-FET, n = 15) and D5 (implantation window, n = 18) post-ovulation. Yellow arrowheads: microvilli; red arrowheads: pinopodes. Scale bars: 20 μ m (low magnification), 10 μ m (high magnification) **(A)**. Proteomic profiling of D3 endometrial tissues (Le: n = 5, LeH: n = 6, NC: n = 5) using 440-protein arrays, with GO and KEGG enrichment analysis **(B–D)**. Metascape analysis of up- and downregulated DEPs **(E,F)**. Venn diagram of shared signaling pathways **(G)**. Sankey diagram of group-DEP-pathway relationships **(H)**. STRING PPI networks for: Le vs. NC (downregulated DEPs), LeH vs. NC (upregulated DEPs), and LeH vs. Le (upregulated DEPs) **(I–K)**.

While no differences were observed in endometrial morphology (Supplementary Figure S1), ultrastructural analysis revealed profound differences. On D5 (the presumed window of implantation), a significantly higher proportion of endometrial samples in the LeH group (84%) exhibited a fully developed receptive phenotype with mature pinopodes, compared to the Le (28%) and NC (60%) groups (Figure 2A; Supplementary Figure S2; Table 3).

**TABLE 3:** Statistics of ultrastructure images from endometrial epithelial tissues.

Period of pinopodes	The $3^{\text{rd}}$ day after ovulation			The $5^{\text{th}}$ day after ovulation		
	NC	Le	LeH	NC	Le	LeH
	(n = 5)	(n = 4)	(n = 6)	(n = 5)	(n = 7)	(n = 6)
Before pinopodes develop	0% (0/5)	0% (0/4)	67% (4/6)	20% (1/5)	14% (1/7)	0% (0/6)
Developing pinopodes	40% (2/5)	50% (2/4)	0% (0/6)	0% (0/5)	14% (1/7)	17% (1/6)
Developing and fully developed pinopodes	0% (0/5)	25% (1/4)	17% (1/6)	0% (0/5)	14% (1/7)	17% (1/6)
Fully developed pinopodes	0% (0/5)	0% (0/4)	0% (0/6)	60% (3/5)	14% (1/7)	67% (4/6)
Regressing pinopodes	60% (3/5)	25% (1/4)	17% (1/6)	20% (1/5)	43% (3/7)	0% (0/6)

NC, natural cycle; Le, Letrozole monotherapy; LeH, low-dose letrozole plus HMG.

In addition, serum hormone analysis indicated that the Le group had significantly lower estradiol levels on D3 relative to both the NC and LeH groups, with no significant differences observed in other hormones (Supplementary Figure S3). Collectively, these results demonstrate that the LeH regimen effectively promotes optimal ultrastructural maturation of the endometrium, facilitating a receptive state for embryo implantation.

### 3.3 LeH promotes endometrial receptivity by upregulating critical signaling pathways

To elucidate the molecular mechanisms by which the LeH regimen enhances endometrial receptivity, we performed protein microarray analysis on endometrial tissues collected on D3 (Supplementary Figures S4, S5). Compared to the NC group, the Le group exhibited a predominant downregulation of DEPs, whereas the LeH group showed primarily upregulation of DEPs (Figure 4A).

Bioinformatic analysis (GO and KEGG) indicated that letrozole monotherapy (Le vs. NC) significantly downregulated pathways critical for implantation, including *Cytokine–cytokine receptor interaction*, *Cell–cell adhesion*, inflammatory processes (e.g., *Rheumatoid arthritis*, *Leukocyte activation*), and pathways related to endometrial development (e.g., *Morphogenesis of an epithelial fold*, *Tube morphogenesis*) (Figure 2B). In contrast, the LeH regimen markedly upregulated these same categories of pathways (Figures 2C,D). Notably, LeH restored the expression of 29 DEPs that were downregulated in the Le group (Figure 4D) and significantly enriched pathways supportive of endometrial development (Figure 2D).

Further enrichment analysis across the three groups revealed that pathways such as *Cell adhesion molecules* and *Positive regulation of response to external stimulus*—which were downregulated in the Le group—were upregulated under the LeH regimen (Figures 2E–G). Sankey analysis identified key DEPs within these pathways, including CD6, CDH1, NCAM1, and SDC3 in cell adhesion, and BMPR2, CCL24, IL21, IL6R, KDR, LAG3, PGC, TLR4, VEGF, and XCL1 in the response to external stimulus (Figure 2H). PPI network analysis demonstrated functional connectivity among these DEPs across comparison groups (Figures 2I–K). Together, these data indicate that the LeH regimen enhances endometrial receptivity by activating a synergistic network of pathways essential for embryo attachment and implantation.

### 3.4 LeH fosters an anti-inflammatory uterine cavity microenvironment

**FIGURE 3:**
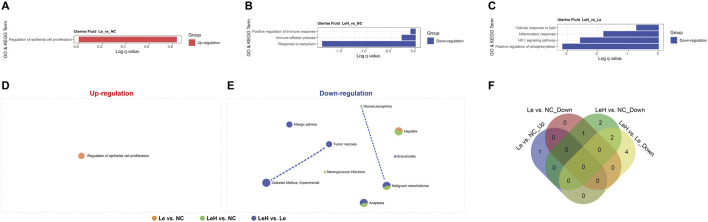
Proteomic profiling of preimplantation uterine fluid. Uterine fluid from Le (n = 4), LeH (n = 6), and NC (n = 4) groups collected on D3 (pre-FET) was analyzed by 440-protein arrays. GO and KEGG enrichment analysis **(A–C)**. Metascape analysis of up- and downregulated DEPs **(D, E)**. Venn diagram of shared pathways **(F)**.

**FIGURE 4:**
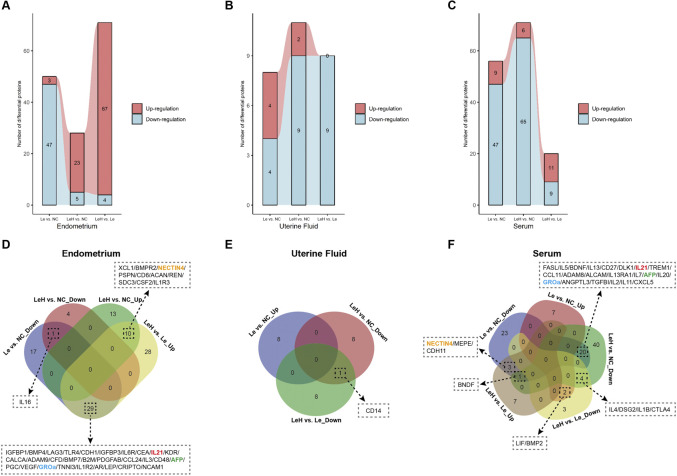
Multi-sample comparison of shared DEPs in endometrial tissues, serum, and uterine fluid at D3 pre-FET. Differential protein expression analysis quantified up- and downregulated proteins across groups and evaluated shared DEPs. This comprehensive assessment was applied to endometrial tissues **(A,D)**, uterine fluid **(B,E)**, and serum **(C,F)**, harvested on day 3 (D3, pre-FET) post-ovulation. Overlapping DEPs are indicated, with identical DEPs shared across sample types represented by the same color.

The uterine fluid, which engages in direct contact with the embryo, plays a crucial role in implantation success. Protein microarray analysis of uterine fluid revealed that, compared to the NC group, the LeH regimen was associated with significant downregulation of processes related to inflammatory responses, including *Response to bacterium* and *Immune effector process* (Figures 3A–C; Figures 4B,E; Supplementary Figures S6, S7). Common pathway enrichment analysis further indicated downregulation of inflammatory processes such as *Hepatitis* in both LeH vs. NC and Le vs. NC comparisons; however, the extent of downregulation was markedly less pronounced in the LeH group (Figures 3D–F). These findings suggest that the LeH regimen promotes an anti-inflammatory and immune-tolerant microenvironment within the uterine fluid, which may protect the embryo from adverse maternal immune reactions.

### 3.5 LeH reduces systemic inflammation, supporting endometrial preparedness

To assess the systemic effects of the LeH regimen, we conducted serum protein microarray analysis. The results indicated a widespread downregulation of DEPs in both the Le and LeH groups compared to the NC group (Figure 4C; Supplementary Figures S8, S9). A total of 20 DEPs were commonly downregulated in the Le and LeH groups relative to NC. Among these, IL21, AFP, and GRO α overlapped with DEPs identified in endometrial tissue. Furthermore, the LeH regimen restored expression levels of three serum DEPs that were significantly suppressed in the Le group, one of which—NECTIN4—was also differentially expressed in the endometrium (Figure 4F).

**FIGURE 5:**
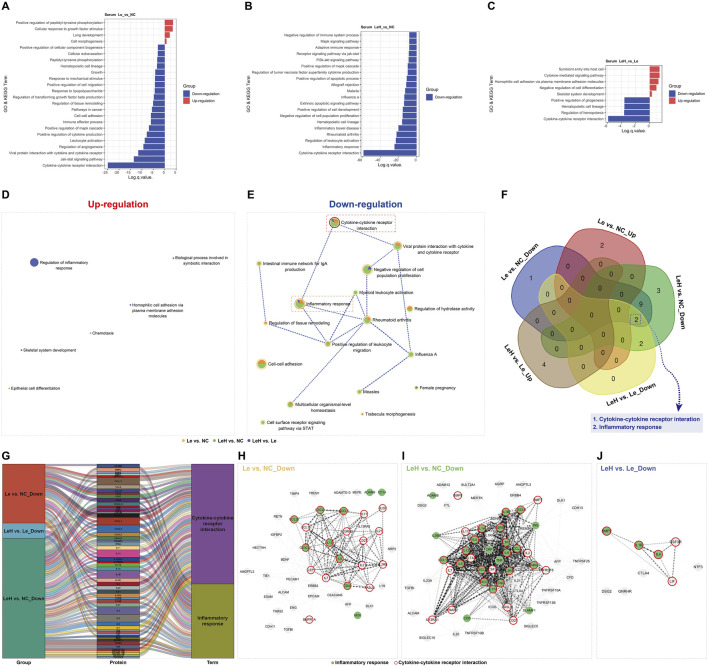
Integrated proteomic profiling of D3 serum with focused pathway analysis of serum DEPs. Proteomic analysis employed a 440-target protein array (11 sub-arrays) on serum samples from groups Le (n = 4), LeH (n = 6), and NC (n = 5), harvested on day 3 (D3, pre-FET) post-ovulation. Bioinformatic analysis included GO and KEGG enrichment bar charts for inter-group comparisons in serum **(A–C)**. Metascape enrichment analysis identified upregulated **(D)** and downregulated **(E)** DEPs in serum, followed by a Venn diagram of shared signaling pathways **(F)**. A Sankey diagram visualized relationships between comparison groups, DEPs, and key signaling pathways **(G)**. Finally, STRING-generated protein–protein interaction networks of DEPs within these key pathways are shown for serum comparisons: Le vs. NC (downregulated DEPs) **(H)**, LeH vs. NC (downregulated DEPs) **(I)**, and LeH vs. Le (downregulated DEPs) **(J)**.

However, the LeH group demonstrated a more pronounced and targeted downregulation of key pathways, most notably *Cytokine–cytokine receptor interaction* and *Inflammatory response* (Figures 5A–F). Interaction network analysis highlighted central roles for cytokines such as CCL11, CXCL1, CXCL5, IL1B, and IL4 in this systemic anti-inflammatory effect (Figures 5G–J). These results suggest that the LeH regimen may enhance endometrial receptivity not only locally but also systemically, through attenuation of pro-inflammatory signals in the circulation.

## 4 Discussion

**FIGURE 6:**
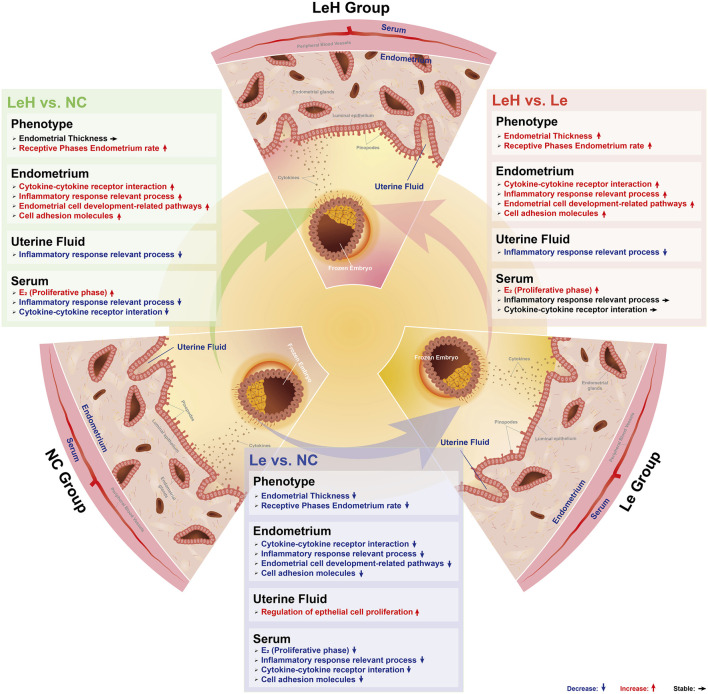
Diagram illustrating the LeH regimen for optimized endometrial preparation in frozen embryo transfer (FET). Compared to NC, the Le group exhibited lower estrogen levels during proliferative/early-secretory phases and fewer patients with receptive-state pinopodes at implantation—potentially due to suppressed endometrial preparation pathways. Conversely, the LeH group showed elevated proliferative-phase estrogen levels, increased receptive pinopodes, and ameliorated systemic inflammation. This enhancement likely arises from LeH-mediated upregulation of endometrial preparation pathways and establishment of an embryo-supportive anti-inflammatory uterine milieu.

This study provides comprehensive clinical and mechanistic evidence supporting the superiority of the LeH regimen in preparing the endometrium for FET. Our findings confirm the initial hypothesis that co-administration of HMG can counteract the negative impact of letrozole monotherapy on endometrial receptivity, ultimately leading to improved pregnancy outcomes. The LeH regimen enhances endometrial receptivity through a multi-faceted mechanism involving structural normalization, molecular pathway activation, and the creation of a supportive local and systemic immune environment (Figure 6).

The ultrastructural observation that the LeH group yielded a significantly higher proportion of endometria with fully developed pinopodes on D5 is a pivotal finding. Although the status of pinopodes as a definitive marker of endometrial receptivity has been debated (Aunapuu et al., 2018; Quinn et al., 2007; Quinn and Casper, 2009; Jin et al., 2017; Qiong et al., 2017), they remain a widely recognized morphological indicator (Lazim et al., 2023; Zhou and Zhou, 2025; Salmasi et al., 2024) whose development is tightly regulated by hormonal cues (Matson et al., 2017; Quinn et al., 2020). The enhanced pinopode development in the LeH group, together with its superior clinical outcomes, suggests that the LeH regimen more effectively establishes the hormonal milieu necessary for a receptive endometrial phenotype. Although our retrospective data featured uneven group sizes, age stratification was employed to control for this potential source of bias.

At the molecular level, our protein array data offer a clear mechanistic explanation for the clinical results. Le group predominantly suppressed the expression of proteins and pathways critical for implantation, including those involved in cell adhesion (Pathare and Hinduja, 2022; Grund and Grümmer, 2018), cytokine signaling, and inflammatory responses (Robertson et al., 2018; Granot et al., 2012). This suppression likely underlies the suboptimal receptivity associated with letrozole monotherapy. Crucially, the LeH regimen not only reversed this suppressive effect but actively upregulated these essential pathways. The restoration of “*Cell adhesion molecules*” and “*P. regulation of response to external stimulus*” pathways is particularly significant, as these processes are fundamental to embryo attachment and stromal decidualization. The identification of key proteins like CDH1, NCAM1, VEGF, and the novel candidate NECTIN4 provides specific therapeutic targets and underscores LeH’s ability to activate a synergistic receptivity network, although the causal relationship requires future validation.

The hormonal profile observed in the LeH group provides a foundational explanation for its benefits (Shaw et al., 2010). The significantly higher estradiol levels on the day of HCG administration, attributable to HMG supplementation, are critical for overcoming the systemic estrogen suppression caused by letrozole. Estradiol is a master regulator of endometrial proliferation, angiogenesis, and the expression of implantation mediators (Robertson et al., 2018; Granot et al., 2012). Thus, by ensuring adequate estrogenic priming, the LeH regimen creates a permissive environment for the subsequent molecular and structural changes that define receptivity, without compromising ovulation or luteal phase progesterone function.

Beyond the endometrium itself, our study reveals that the LeH regimen favorably modulates the uterine cavity (Sang et al., 2020; Zhang et al., 2017) and systemic environments. The anti-inflammatory signature observed in the uterine fluid of the LeH group—characterized by the downregulation of *immune effector processes*—suggests a shift towards immune tolerance, which is vital for protecting the semi-allogeneic embryo (Clark et al., 2015; Pantos et al., 2021). Furthermore, the pronounced downregulation of systemic inflammatory pathways, such as “*Cytokine-cytokine receptor interaction*,” indicates that LeH benefits the endometrium not only directly but also remotely by reducing a potentially harmful circulating inflammatory state, which is known to be detrimental to implantation and pregnancy maintenance (Li et al., 2024; Zhang H. et al., 2023).

## 5 Conclusion

In conclusion, our results demonstrate that the LeH regimen represents an optimized approach for endometrial preparation in FET cycles. It successfully mitigates the principal drawback of letrozole—impaired endometrial receptivity due to hypoestrogenism—by providing supplemental gonadotropin support. The regimen acts through a coordinated hierarchy of effects: (1) ensuring sufficient estradiol levels for endometrial proliferation and priming; (2) upregulating key molecular pathways governing embryo adhesion and implantation; (3) fostering a receptive endometrial ultrastructure; and (4) inducing a protective anti-inflammatory environment both locally within the uterus and systemically. Future prospective randomized studies are warranted to confirm these findings and to refine patient selection criteria for this promising protocol.

## Data Availability

The data that support the findings of this study are available from the corresponding author upon reasonable request.
