# Supplementary material for: Low-dose letrozole-HMG regimen reverses letrozole-induced endometrial impairment and improves frozen embryo transfer outcomes
Source: Front Cell Dev Biol. 2025 Dec 16;13:1725350. doi: 10.3389/fcell.2025.1725350 (PMC12748246; doi:10.3389/fcell.2025.1725350)
Supplement: Supplementary file 1 [file DataSheet1.pdf]

Supplementary Material

1 SUPPLEMENTARY FIGURES

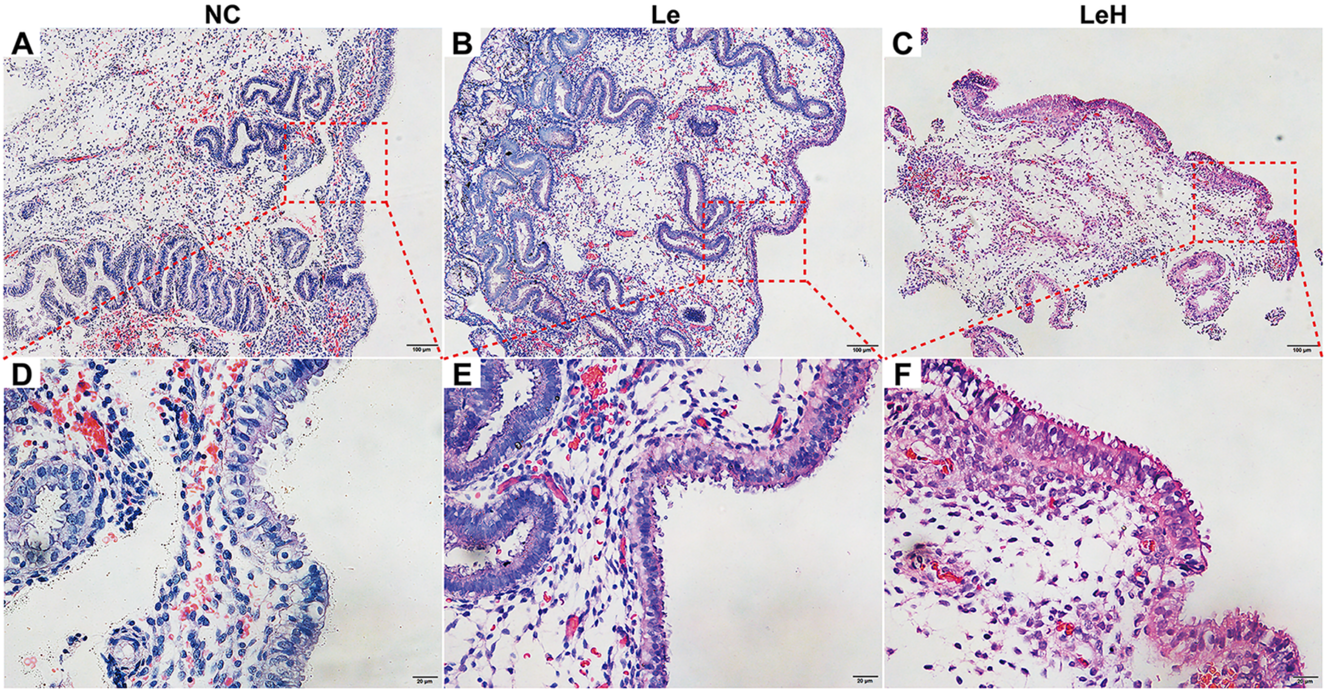

Figure S1. Endometrial morphology analysis. Endometrial tissues stained with H&E in three groups: NC (A, D), Le (B, E), and LeH (C, F). Top row: low-magnification morphology (scale bars = 100 μm); bottom row: high-magnification views (scale bars = 20 μm).

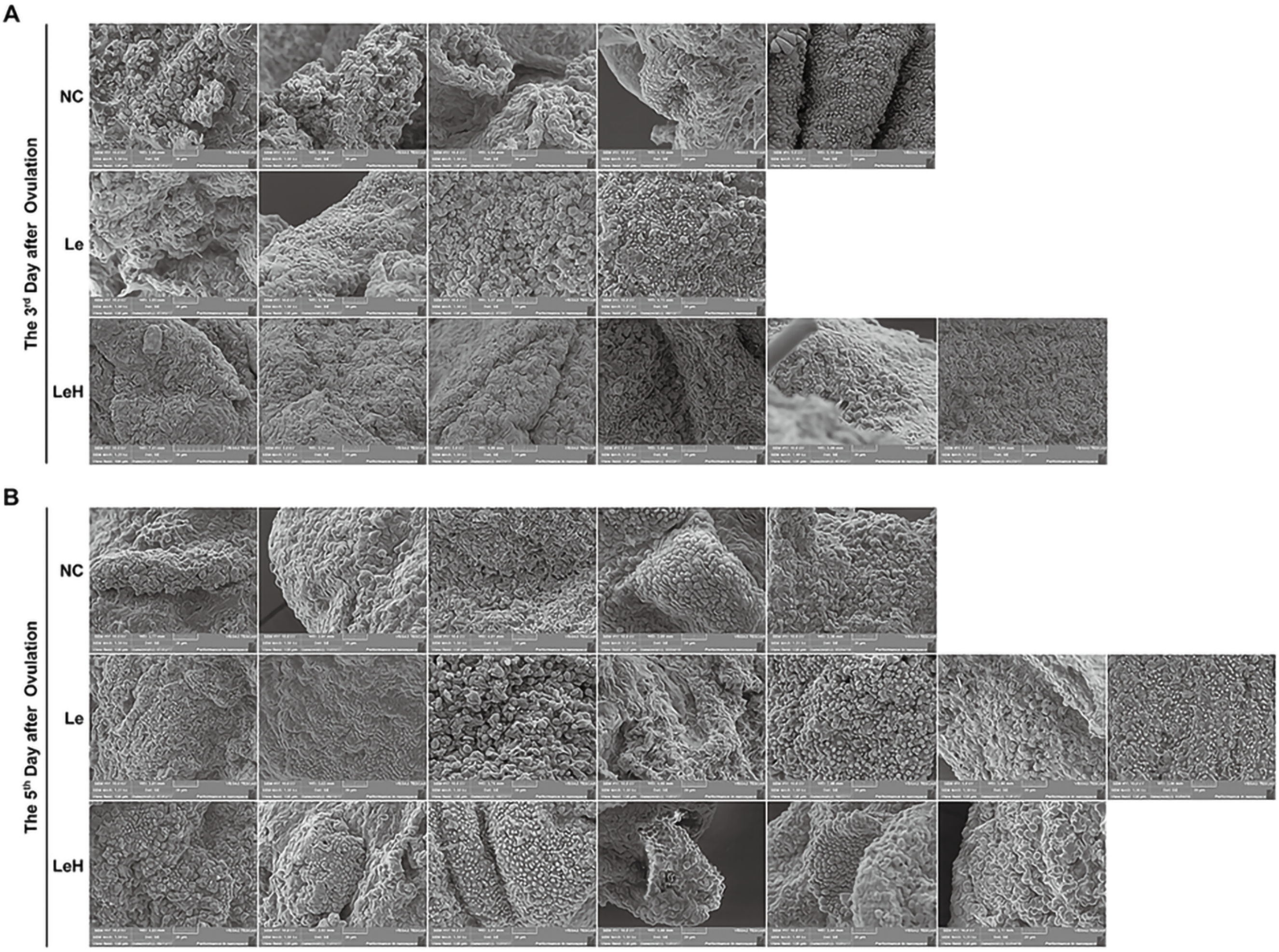

Figure S2. Endometrial scanning electron microscopy (SEM) analysis. Apical surfaces of endometrial epithelia examined by SEM on post-ovulatory day 3 (A) and day 5 (B). Groups: NC (n = 10), Le (n = 11), LeH (n = 12).

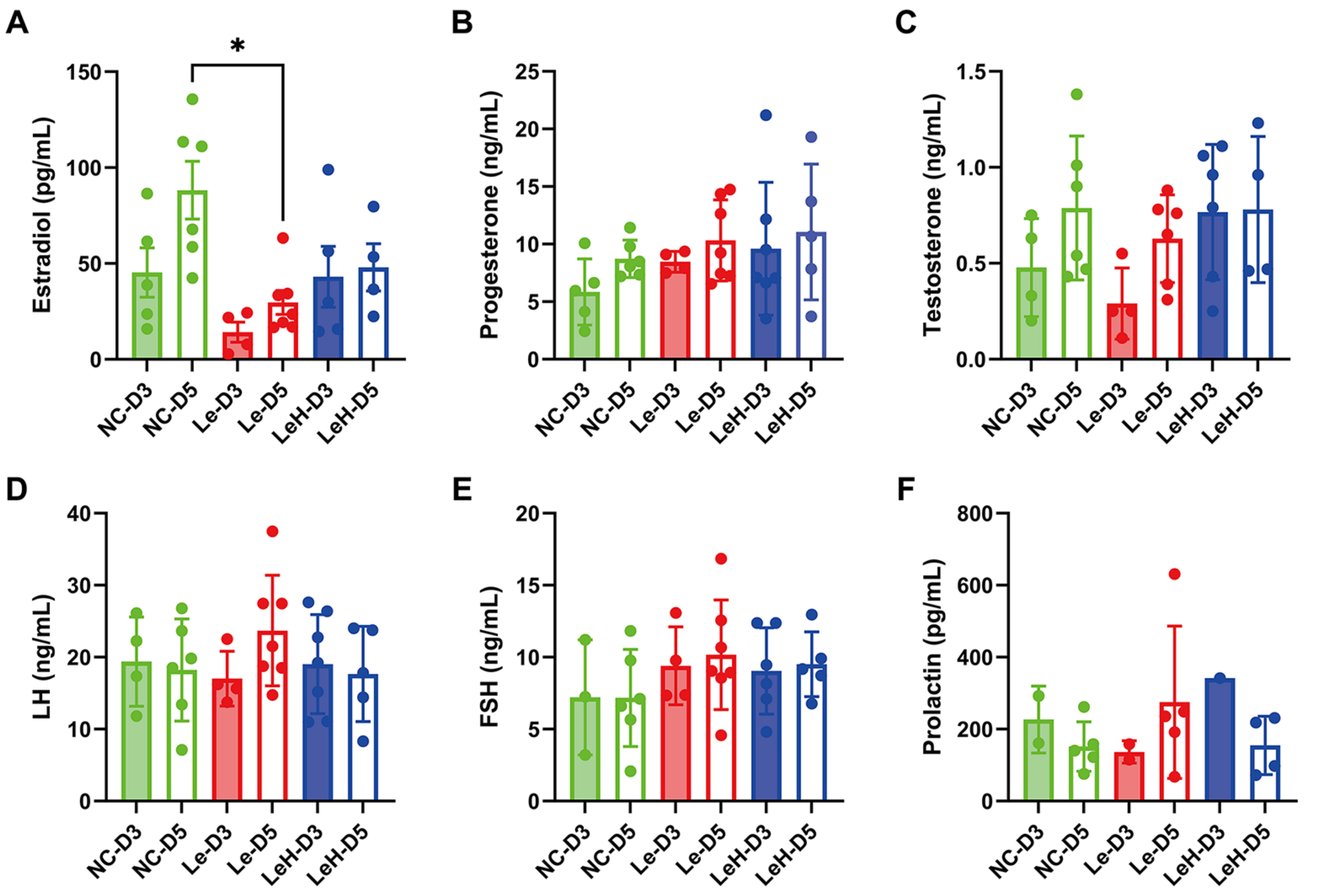

Figure S3. Serum hormone levels on post-ovulatory day 3 and 5. Serum levels of Estradiol (A), Progesterone (B), Testosterone (C), LH (D), FSH (E), and Prolactin (F) measured on the 3rd (D3) and 5th (D5) day following ovulation. Groups: NC (n = 11), Le (n = 11), LeH (n = 12).

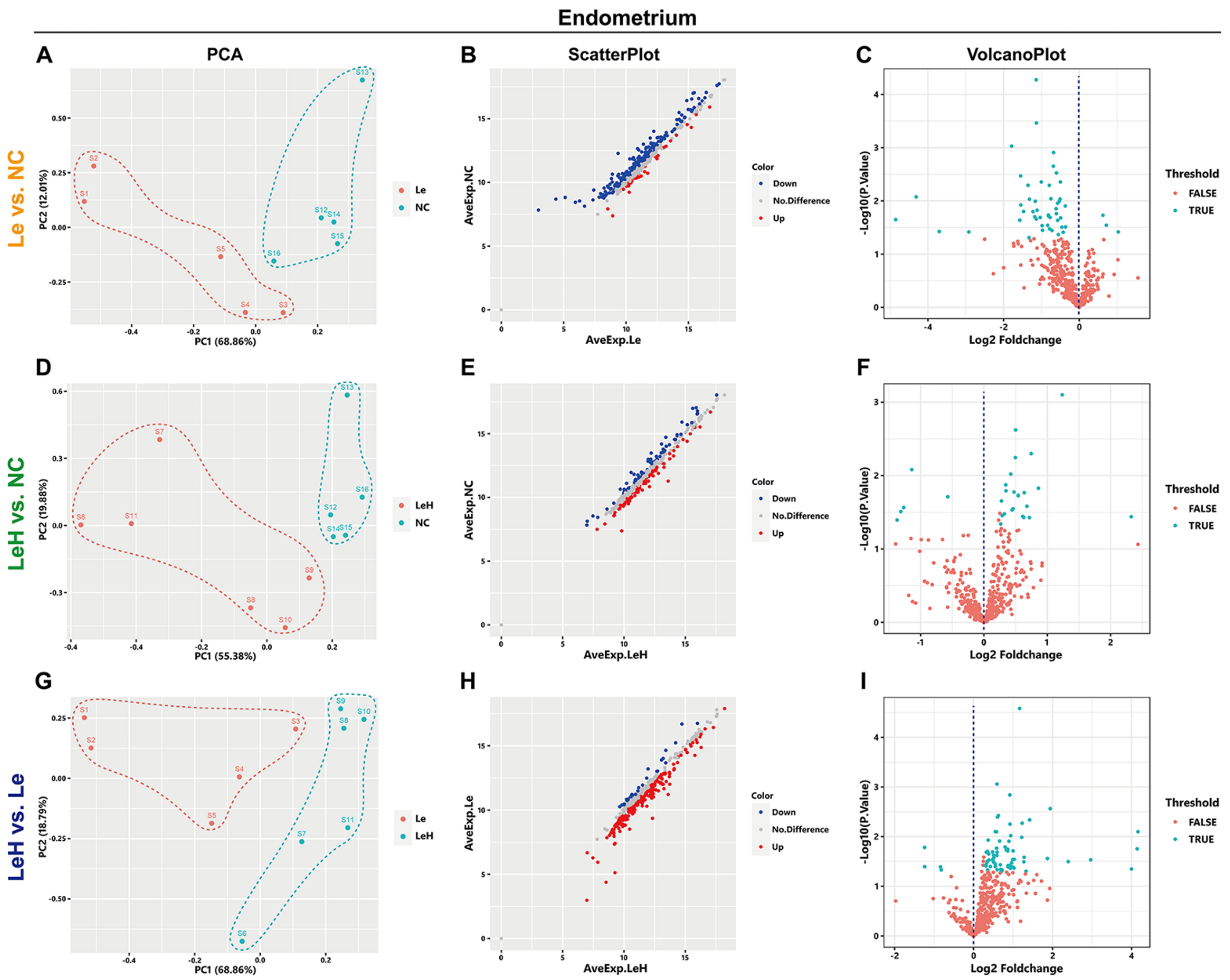

Figure S4. Endometrial proteomic analysis using protein arrays. Endometrial tissues (day 3 post-ovulation) from Le (n = 5), LeH (n = 6), and NC (n = 5) groups analyzed via 440-protein arrays. Bioinformatics comparisons: Le vs. NC (A-C), LeH vs. NC (D-F), LeH vs. Le (G-I). PCA, ScatterPlots, and VolcanoPlots shown.

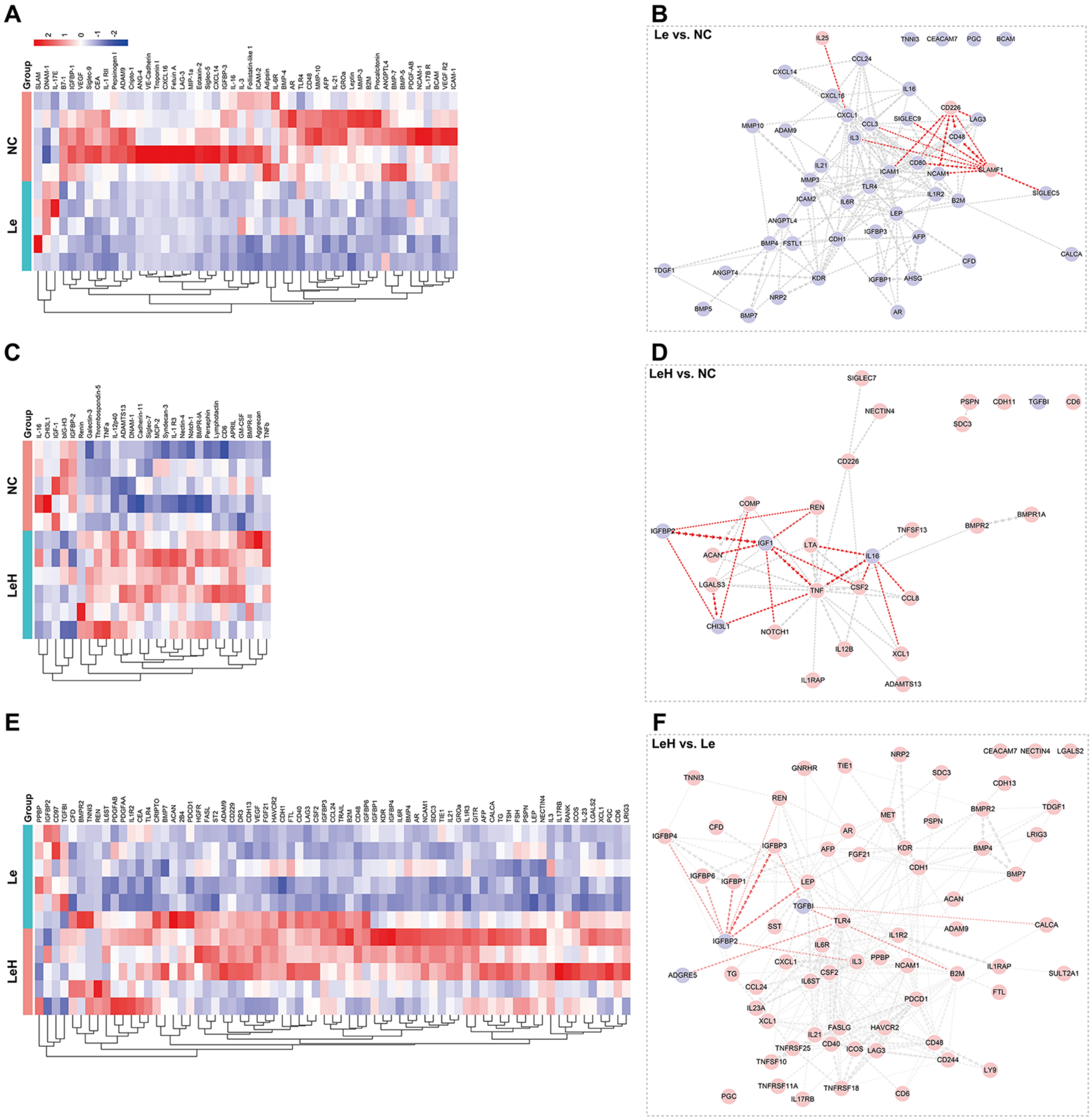

Figure S5. Heatmaps and PPI networks of endometrial proteomes. Endometrial tissues (day 3 post-ovulation) from Le (n = 5), LeH (n = 6), and NC (n = 5) groups analyzed via 440-protein arrays. Heatmaps and protein-protein interaction (PPI) networks for Le vs. NC (A-C), LeH vs. NC (D-F), LeH vs. Le (G-I).

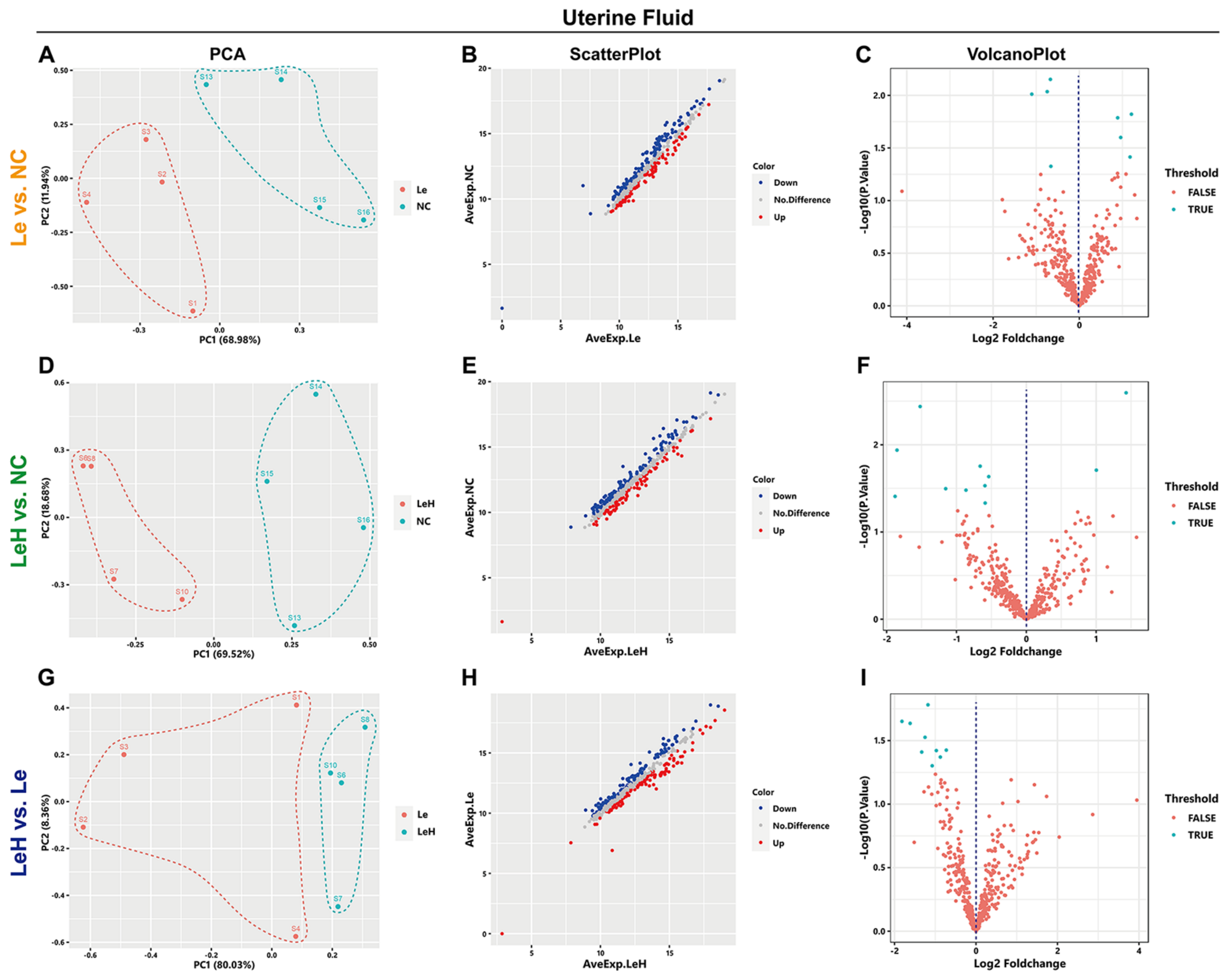

Figure S6. Uterine fluid proteomic analysis using protein arrays. Uterine fluid (day 3 post-ovulation) from Le (n = 4), LeH (n = 6), and NC (n = 4) groups analyzed via 440-protein arrays. Bioinformatics comparisons: Le vs. NC (A-C), LeH vs. NC (D-F), LeH vs. Le (G-I). PCA, ScatterPlots, and VolcanoPlots shown.

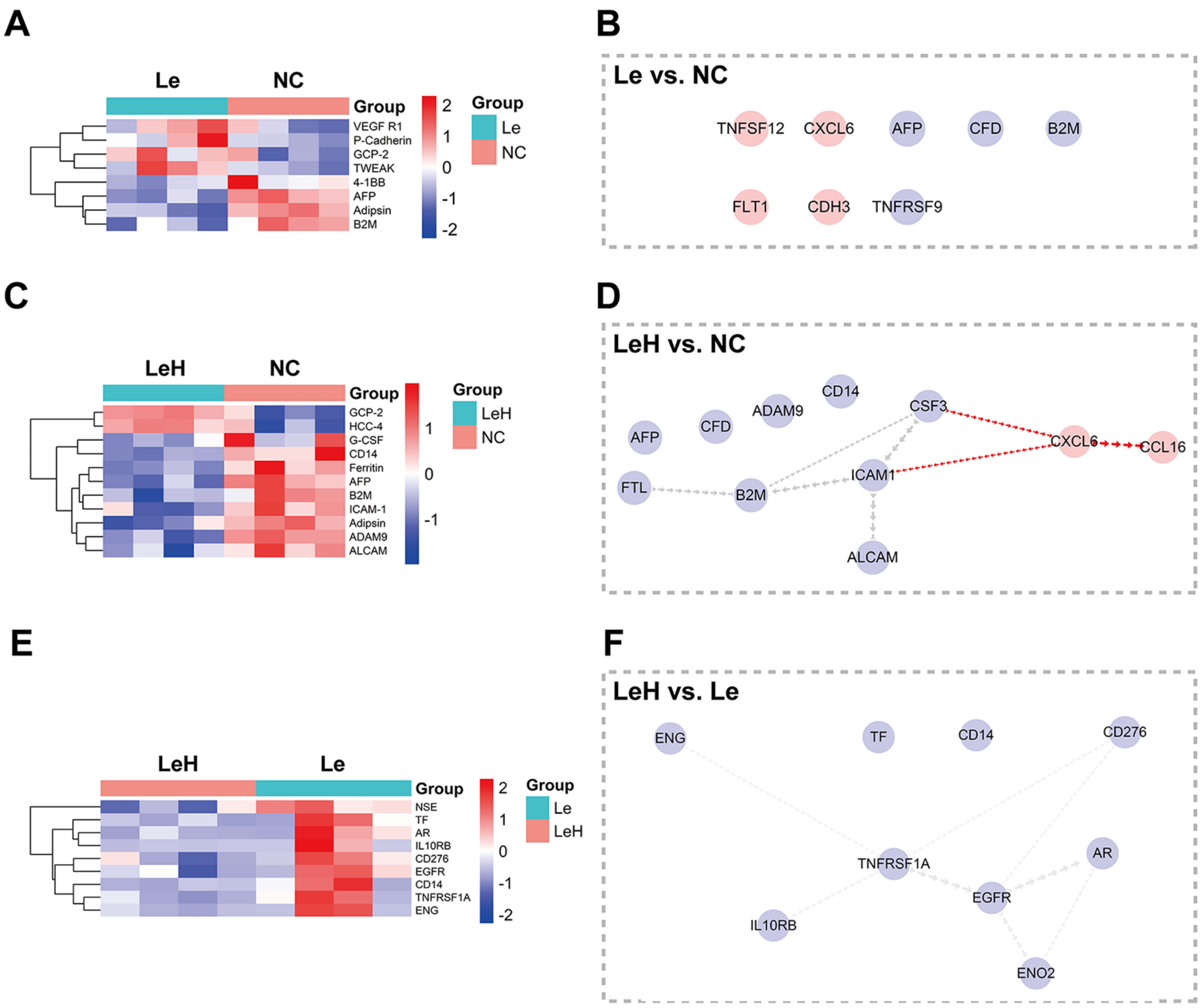

Figure S7. Heatmaps and PPI networks of uterine fluid proteomes. Uterine fluid (day 3 post-ovulation) from Le (n = 4), LeH (n = 6), and NC (n = 4) groups. Heatmaps and PPI networks for Le vs. NC (A-C), LeH vs. NC (D-F), LeH vs. Le (G-I).

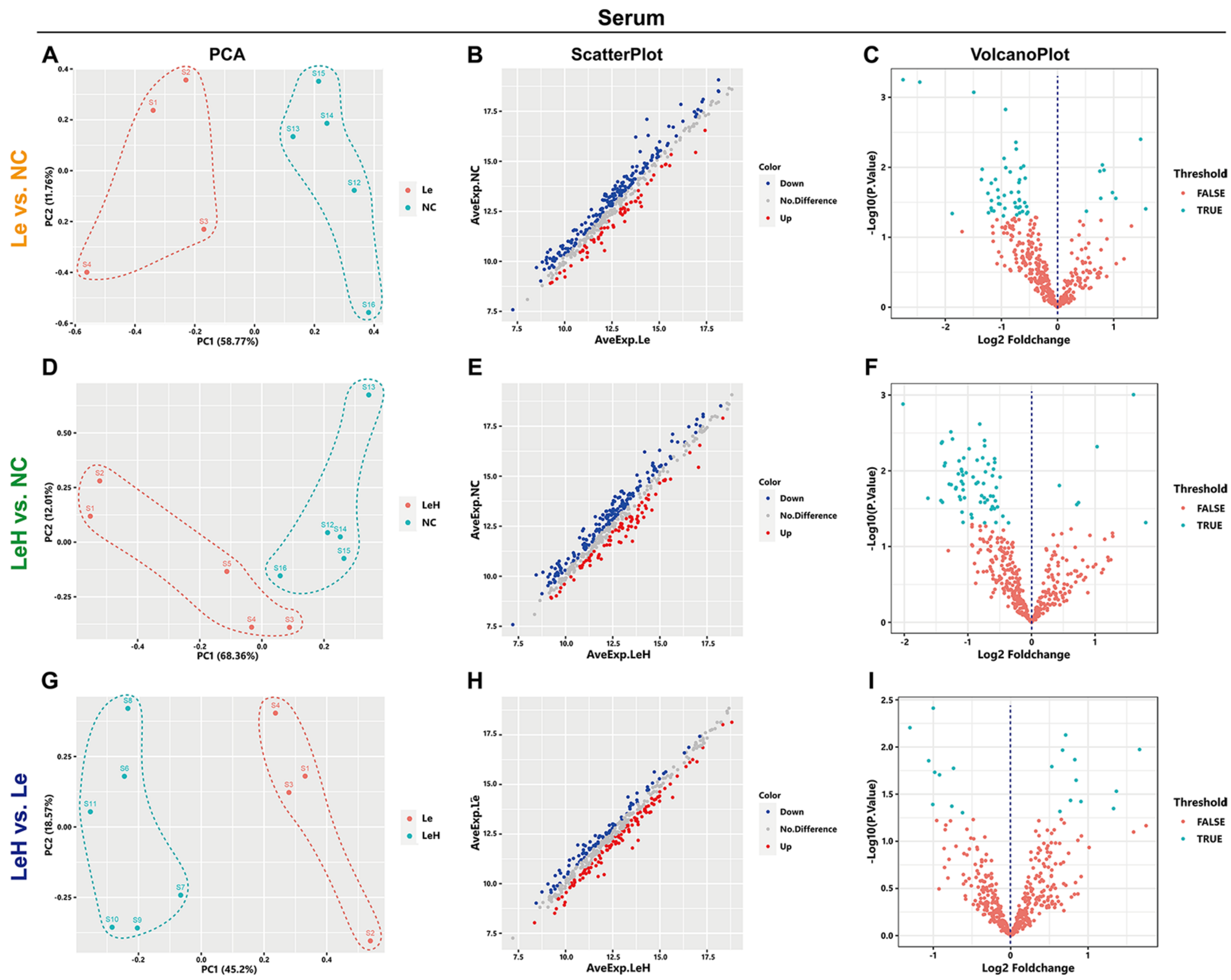

Figure S8. Serum proteomic analysis using protein arrays. Serum samples (day 3 post-ovulation) from Le (n = 4), LeH (n = 6), and NC (n = 5) groups analyzed via 440-protein arrays. Bioinformatics comparisons: Le vs. NC (A-C), LeH vs. NC (D-F), LeH vs. Le (G-I). PCA, ScatterPlots, and VolcanoPlots shown.

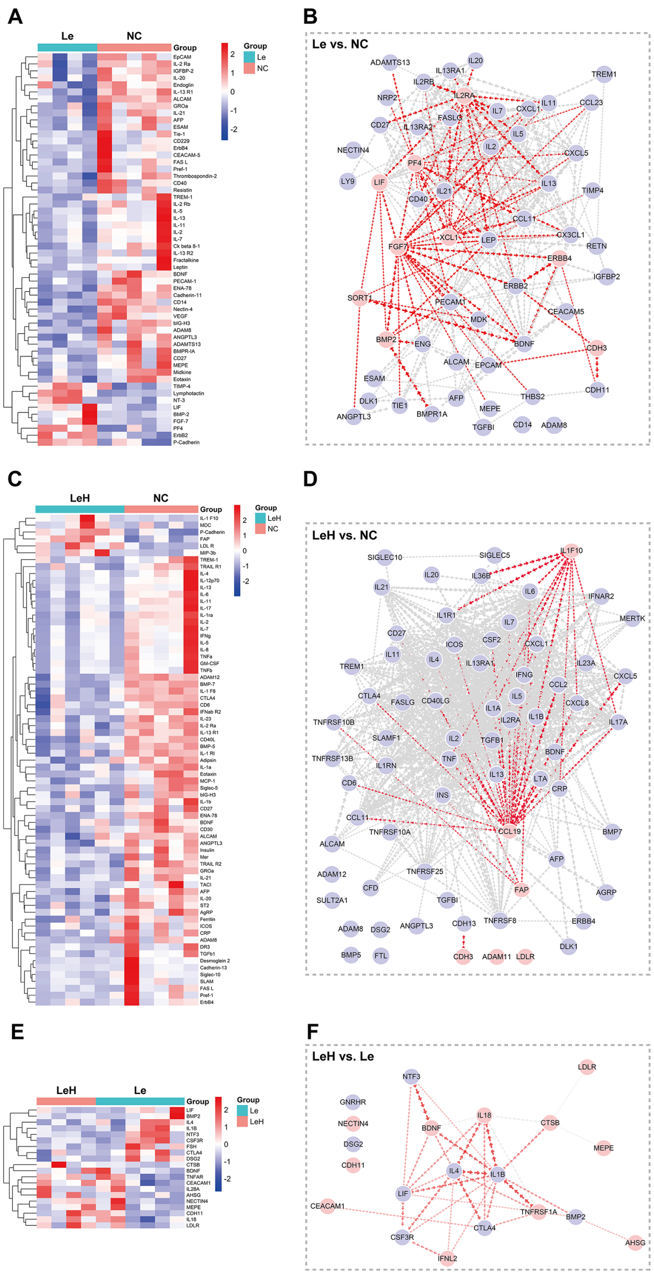

Figure S9. Heatmaps and PPI networks of serum proteomes. Serum (day 3 post-ovulation) from Le (n = 4), LeH (n = 6), and NC (n = 5) groups. Heatmaps and PPI networks for Le vs. NC (A-C), LeH vs. NC (D-F), LeH vs. Le (G-I).
